# 3-Hydr­oxy-*N*′-[(*Z*)-(5-methyl-2-fur­yl)methyl­idene]naphthalene-2-carbo­hydrazide

**DOI:** 10.1107/S1600536809043141

**Published:** 2009-10-23

**Authors:** Zahid Shafiq, Muhammad Yaqub, M. Nawaz Tahir, Mian Hasnain Nawaz, M. Saeed Iqbal

**Affiliations:** a Department of Chemistry, Bahauddin Zakariya University, Multan 60800, Pakistan; b Department of Physics, University of Sargodha, Sargodha, Pakistan; c Department of Chemistry, Government College University, Lahore, Pakistan

## Abstract

The asymmetric unit of title compound, C_17_H_14_N_2_O_3_, contains three independent mol­ecules. In one of these mol­ecules, the 5-methyl-2-furyl group is disordered over two sets of sites with an occupancy ratio of 0.747 (3):0.253 (3). In the two ordered mol­ecules, the furan and naphthalene rings are oriented at dihedral angles of 11.05 (12) and 32.2 (5)°. In the disordered mol­ecule, the furan rings with major and minor occupancies are oriented at dihedral angles of 41.4 (2) and 26.6 (13)°, respectively, with the corresponding naphthalene ring. An intra­molecular O—H⋯O hydrogen bond occurs within each mol­ecule. In the crystal, mol­ecules are linked by N—H⋯O, N—H⋯(N,O) and C—H⋯O inter­actions.

## Related literature

For related structures, see: Bai & Jing (2007 ▶); Huang (2009 ▶); Liu & Li (2004 ▶); Shafiq *et al.* (2009*a* ▶,*b* ▶); Yao & Jing (2007 ▶). For graph-set motifs, see: Bernstein *et al.* (1995 ▶).
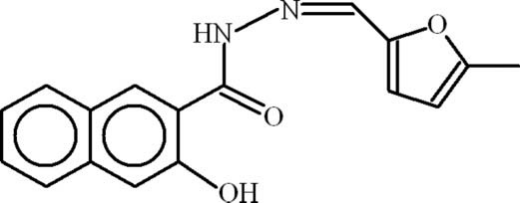


## Experimental

### 

#### Crystal data

C_17_H_14_N_2_O_3_
*M* *_r_* = 294.30
Triclinic, 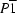

*a* = 10.604 (4) Å
*b* = 12.321 (5) Å
*c* = 18.615 (3) Å
α = 71.351 (5)°
β = 73.742 (4)°
γ = 84.280 (5)°
*V* = 2212.1 (13) Å^3^
*Z* = 6
Mo *K*α radiation
μ = 0.09 mm^−1^
*T* = 296 K
0.25 × 0.20 × 0.18 mm

#### Data collection

Bruker Kappa APEXII CCD diffractometer
Absorption correction: multi-scan (*SADABS*; Bruker, 2005 ▶) *T*_min_ = 0.975, *T*_max_ = 0.985
48594 measured reflections
11199 independent reflections
5268 reflections with *I* > 2σ(*I*)
*R*_int_ = 0.044

#### Refinement

*R*[*F* ^2^ > 2σ(*F* ^2^)] = 0.048
*wR*(*F* ^2^) = 0.130
*S* = 0.99
11199 reflections
624 parameters
7 restraints
H-atom parameters constrained
Δρ_max_ = 0.16 e Å^−3^
Δρ_min_ = −0.16 e Å^−3^

### 

Data collection: *APEX2* (Bruker, 2007 ▶); cell refinement: *SAINT* (Bruker, 2007 ▶); data reduction: *SAINT*; program(s) used to solve structure: *SHELXS97* (Sheldrick, 2008 ▶); program(s) used to refine structure: *SHELXL97* (Sheldrick, 2008 ▶); molecular graphics: *ORTEP-3 for Windows* (Farrugia, 1997 ▶) and *PLATON* (Spek, 2009 ▶); software used to prepare material for publication: *WinGX* (Farrugia, 1999 ▶) and *PLATON* (Spek, 2009 ▶).

## Supplementary Material

Crystal structure: contains datablocks global, I. DOI: 10.1107/S1600536809043141/hb5157sup1.cif

Structure factors: contains datablocks I. DOI: 10.1107/S1600536809043141/hb5157Isup2.hkl

Additional supplementary materials:  crystallographic information; 3D view; checkCIF report

## Figures and Tables

**Table 1:** Hydrogen-bond geometry (Å, °)

*D*—H⋯*A*	*D*—H	H⋯*A*	*D*⋯*A*	*D*—H⋯*A*
N2—H2*N*⋯O8^i^	0.86	2.15	2.869 (2)	142
N4—H4*N*⋯O2	0.86	2.20	2.896 (2)	137
N6—H6*N*⋯O5^ii^	0.86	2.36	3.074 (2)	140
N6—H6*N*⋯N3^ii^	0.86	2.44	3.212 (2)	149
O3—H3*O*⋯O2	0.82	1.89	2.612 (3)	145
O6—H6*O*⋯O5	0.82	1.89	2.603 (2)	145
O9—H9*O*⋯O8	0.82	1.86	2.588 (2)	147
C34—H34⋯O2	0.93	2.48	3.398 (3)	167
C40—H40⋯O5^ii^	0.93	2.42	3.185 (3)	140
C44—H44⋯O1*A*	0.93	2.59	3.514 (5)	176
C48—H48⋯O3^ii^	0.93	2.51	3.149 (3)	126

Symmetry codes: (i) 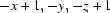; (ii) 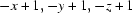.

**Fig. 1.**
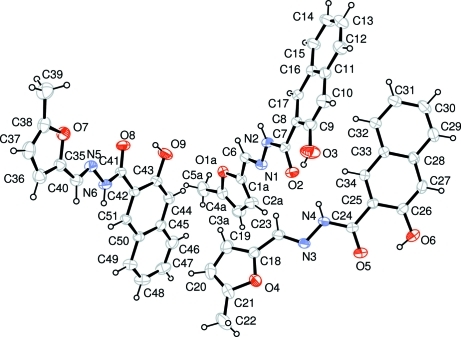
View of (I) with the atom numbering scheme for atoms of greater occupancy. The displacement ellipsoids are drawn at the 30% probability level. H-atoms are shown by small circles of arbitrary radii.

**Fig. 2.**
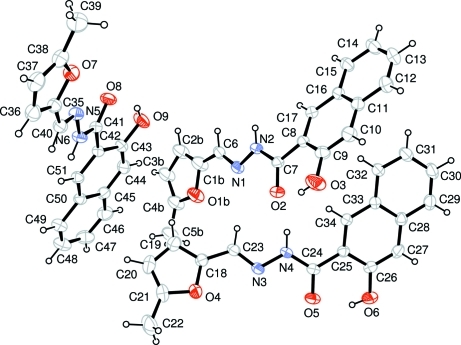
View of (I) with the atom numbering scheme for atoms of smaller occupancy ratio. The displacement ellipsoids are drawn at the 30% probability level. H-atoms are shown by small circles of arbitrary radii.

**Fig. 3.**
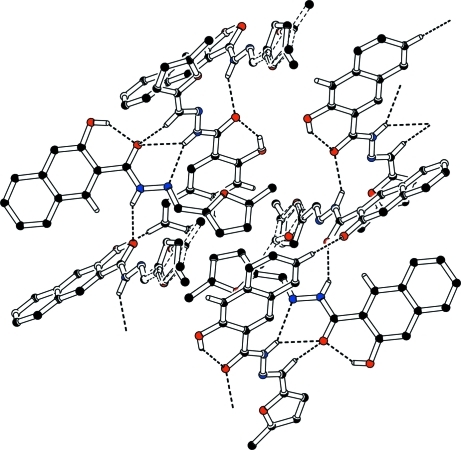
The partial packing for (I), which shows that molecules form polymeric chains and have different ring motifs. H-atoms not involved in H-bondings have been omitted for clarity.

## supplementary crystallographic information

### Comment

In continuation to the formation of different hydrazide derivatives (Shafiq *et al.*, 2009*a*, 2009*b*), the title compound (I, Fig. 1), has been prepared and being reported.

The crystal structures of (II) 2-Hydroxyacetophenone 3-hydroxy-2-naphthoylhydrazone (Liu & Li, 2004), (III) *N*'-[4-(Dimethylamino)benzylidene]-3-hydroxy-2-naphthohydrazide (Huang, 2009), (IV) (*E*)-*N*'-((5-Methylfuran-2-yl)methylene)furan-2-carbohydrazide (Yao & Jing, 2007) and (V) (*E*)-4-Bromo-*N*'-((5-methylfuran-2-yl)methylene)benzohydrazide (Bai & Jing, 2007) have been published which contain both of the moieties involved in (I).

There are three molecules of (I) in the asymmetric unit of title compound. All differ from one another. This difference is observed from the dihedral angles between the rings and also from disorder. In the disordered molecule the furan and naphthalene group A (C1A–C4A/O1A) and B (C8–C17), respectively are oriented at a dihedral angle of 41.4 (2)°. In the molecule containing O4, the groups C (C18–C22/O4) and D (C25–C34) make a dihedral angle of 32.2 (5)°. Similarly in the remaining molecule, the groups E (C35–C38/O7) and F (C42–C51) are oriented at a dihedral angle of 11.05 (12)°. The dihedral angle between the disordered rings [A (C1A–C4A/O1A) and G (C1B–C4B/O1B)] of furan is 15 (2)°. The molecules are stabilized in the form of infinite one dimensional polymeric chains extending along the crystallographic *b* axis and also there exists various ring motifs (Fig. 2) (Bernstein *et al.*, 1995).

### Experimental

To a hot stirred solution of 3-hydroxy-2-naphthoichydrazide (1.5 g, 7.4 mmol) in ethanol (35 ml) and 1,4-dioxan (2 ml) was added 5-methylfurfural (0.736 ml, 7.4 mmol). The resultant mixture was then heated under reflux. After 30 minutes solid product began to form. The reaction mixture was refluxed about 1.5 h for the sake of completion of reaction which was monitored through TLC. The mixture was cooled to room temperature and the solid was collected by suction filtration. The precipitates were washed with 1,4-dioxan, filtered and dried. Yellow prisms of (I) were obtained by recrystallization of the crude product in 1,4-dioxan after five days.

### Refinement

The disorder in one of the 5-methylfuran was detected through higher values of thermal parameters and from residual peaks. To overcome the large or small bond distances *DFIX* was utilized during refinement. The group containing lower occupancy factor was refined using EADP.

The H-atoms were positioned geometrically (O–H = 0.82 Å, N–H = 0.86 Å, C–H = 0.93–0.96 Å) and refined as riding with *U*_iso_(H) = 1.2*U*_eq_(carrier) or 1.5*U*_eq_(methyl C).

### Crystal data

**Table:**

C_17_H_14_N_2_O_3_	*Z* = 6
*M**_r_* = 294.30	*F*(000) = 924
Triclinic, *P*1	*D*_x_ = 1.326 Mg m^−^^3^
Hall symbol: -P 1	Mo *K*α radiation, λ = 0.71073 Å
*a* = 10.604 (4) Å	Cell parameters from 5268 reflections
*b* = 12.321 (5) Å	θ = 2.4–28.7°
*c* = 18.615 (3) Å	µ = 0.09 mm^−^^1^
α = 71.351 (5)°	*T* = 296 K
β = 73.742 (4)°	Prisms, yellow
γ = 84.280 (5)°	0.25 × 0.20 × 0.18 mm
*V* = 2212.1 (13) Å^3^	

### Data collection

**Table:**

Bruker Kappa APEXII CCD diffractometer	11199 independent reflections
Radiation source: fine-focus sealed tube	5268 reflections with *I* > 2σ(*I*)
graphite	*R*_int_ = 0.044
Detector resolution: 7.40 pixels mm^-1^	θ_max_ = 28.7°, θ_min_ = 2.4°
ω scans	*h* = −14→14
Absorption correction: multi-scan (*SADABS*; Bruker, 2005)	*k* = −16→16
*T*_min_ = 0.975, *T*_max_ = 0.985	*l* = −24→24
48594 measured reflections	

### Refinement

**Table:**

Refinement on *F*^2^	Primary atom site location: structure-invariant direct methods
Least-squares matrix: full	Secondary atom site location: difference Fourier map
*R*[*F*^2^ > 2σ(*F*^2^)] = 0.048	Hydrogen site location: inferred from neighbouring sites
*wR*(*F*^2^) = 0.130	H-atom parameters constrained
*S* = 0.99	*w* = 1/[σ^2^(*F*_o_^2^) + (0.0516*P*)^2^ + 0.1613*P*] where *P* = (*F*_o_^2^ + 2*F*_c_^2^)/3
11199 reflections	(Δ/σ)_max_ = 0.001
624 parameters	Δρ_max_ = 0.16 e Å^−^^3^
7 restraints	Δρ_min_ = −0.16 e Å^−^^3^

### Special details

**Table:**

Geometry. Bond distances, angles *etc*. have been calculated using the rounded fractional coordinates. All su's are estimated from the variances of the (full) variance-covariance matrix. The cell e.s.d.'s are taken into account in the estimation of distances, angles and torsion angles
Refinement. Refinement of *F*^2^ against ALL reflections. The weighted *R*-factor *wR* and goodness of fit *S* are based on *F*^2^, conventional *R*-factors *R* are based on *F*, with *F* set to zero for negative *F*^2^. The threshold expression of *F*^2^ > σ(*F*^2^) is used only for calculating *R*-factors(gt) *etc*. and is not relevant to the choice of reflections for refinement. *R*-factors based on *F*^2^ are statistically about twice as large as those based on *F*, and *R*- factors based on ALL data will be even larger.

### Fractional atomic coordinates and isotropic or equivalent isotropic displacement parameters (Å^2^)

**Table:**

	*x*	*y*	*z*	*U*_iso_*/*U*_eq_	Occ. (<1)
O1A	0.9959 (4)	0.1541 (3)	0.4542 (3)	0.0562 (9)	0.747 (3)
O2	0.65580 (14)	0.43845 (9)	0.24178 (8)	0.0631 (5)	
O3	0.47122 (17)	0.46866 (13)	0.16900 (11)	0.0921 (7)	
N1	0.80165 (15)	0.29221 (12)	0.32581 (9)	0.0531 (6)	
N2	0.73255 (15)	0.25872 (12)	0.28429 (8)	0.0510 (5)	
C1A	0.9445 (6)	0.2436 (3)	0.4053 (3)	0.0483 (11)	0.747 (3)
C2A	0.9913 (5)	0.3407 (4)	0.4028 (3)	0.0636 (16)	0.747 (3)
C3A	1.0743 (4)	0.3126 (4)	0.4533 (4)	0.0673 (16)	0.747 (3)
C4A	1.0758 (8)	0.1983 (6)	0.4837 (4)	0.0562 (11)	0.747 (3)
C5A	1.1352 (3)	0.1156 (3)	0.54123 (18)	0.0815 (12)	0.747 (3)
C6	0.86051 (19)	0.21126 (16)	0.36670 (11)	0.0541 (7)	
C7	0.66876 (18)	0.33839 (14)	0.23901 (10)	0.0476 (6)	
C8	0.61847 (17)	0.30478 (13)	0.18306 (10)	0.0454 (6)	
C9	0.52377 (19)	0.37537 (15)	0.14756 (12)	0.0569 (7)	
C10	0.4845 (2)	0.35028 (16)	0.09087 (12)	0.0631 (8)	
C11	0.53739 (18)	0.25684 (15)	0.06467 (11)	0.0540 (7)	
C12	0.5025 (2)	0.23053 (19)	0.00403 (12)	0.0708 (8)	
C13	0.5589 (3)	0.1406 (2)	−0.02024 (13)	0.0818 (10)	
C14	0.6522 (3)	0.07105 (19)	0.01321 (13)	0.0792 (10)	
C15	0.6889 (2)	0.09305 (16)	0.07126 (12)	0.0636 (8)	
C16	0.63285 (18)	0.18610 (14)	0.09851 (10)	0.0472 (6)	
C17	0.67088 (17)	0.21241 (13)	0.15727 (10)	0.0469 (6)	
C1B	0.947 (3)	0.2146 (16)	0.4118 (16)	0.086 (3)	0.253 (3)
C2B	1.014 (3)	0.141 (2)	0.4568 (17)	0.086 (3)	0.253 (3)
C3B	1.071 (3)	0.205 (2)	0.4924 (18)	0.086 (3)	0.253 (3)
C4B	1.035 (2)	0.3134 (17)	0.4682 (16)	0.086 (3)	0.253 (3)
C5B	1.0541 (8)	0.4178 (3)	0.4832 (5)	0.086 (3)	0.253 (3)
O1B	0.9529 (14)	0.3203 (12)	0.4215 (9)	0.086 (3)	0.253 (3)
O4	0.65494 (13)	0.67372 (10)	0.43795 (7)	0.0614 (5)	
O5	0.84414 (14)	0.82850 (10)	0.14400 (8)	0.0702 (5)	
O6	0.95849 (17)	0.89165 (10)	−0.00598 (9)	0.0790 (6)	
N3	0.76595 (14)	0.66539 (12)	0.28104 (9)	0.0485 (5)	
N4	0.80070 (15)	0.64348 (11)	0.20930 (9)	0.0515 (6)	
C18	0.69926 (19)	0.57430 (15)	0.42005 (12)	0.0550 (7)	
C19	0.6813 (2)	0.48682 (18)	0.48745 (13)	0.0751 (9)	
C20	0.6243 (2)	0.5319 (2)	0.54963 (13)	0.0788 (9)	
C21	0.6085 (2)	0.6441 (2)	0.51818 (13)	0.0687 (9)	
C22	0.5512 (3)	0.7378 (2)	0.55129 (15)	0.1067 (11)	
C23	0.74565 (19)	0.57470 (15)	0.34036 (12)	0.0554 (7)	
C24	0.83427 (17)	0.73040 (14)	0.14223 (11)	0.0474 (6)	
C25	0.85989 (16)	0.70440 (13)	0.06697 (10)	0.0442 (6)	
C26	0.92304 (18)	0.78819 (14)	−0.00429 (12)	0.0527 (7)	
C27	0.95030 (19)	0.76484 (15)	−0.07400 (12)	0.0586 (7)	
C28	0.91602 (18)	0.66060 (15)	−0.07890 (11)	0.0510 (7)	
C29	0.9420 (2)	0.63507 (18)	−0.15087 (12)	0.0663 (8)	
C30	0.9066 (2)	0.53385 (19)	−0.15254 (13)	0.0698 (9)	
C31	0.8445 (2)	0.45044 (18)	−0.08347 (13)	0.0650 (8)	
C32	0.81850 (19)	0.47160 (15)	−0.01349 (11)	0.0548 (7)	
C33	0.85240 (17)	0.57673 (14)	−0.00917 (10)	0.0446 (6)	
C34	0.82635 (17)	0.60206 (14)	0.06213 (10)	0.0461 (6)	
O7	−0.09686 (12)	−0.07040 (10)	0.73469 (7)	0.0568 (5)	
O8	0.38574 (13)	−0.03678 (10)	0.64992 (8)	0.0619 (5)	
O9	0.61823 (14)	0.03617 (13)	0.56670 (9)	0.0758 (6)	
N5	0.14612 (15)	0.01505 (11)	0.72496 (9)	0.0499 (5)	
N6	0.25674 (15)	0.07340 (11)	0.71746 (9)	0.0505 (5)	
C35	−0.08637 (19)	0.00704 (14)	0.77153 (11)	0.0508 (7)	
C36	−0.2056 (2)	0.02625 (17)	0.81559 (12)	0.0652 (8)	
C37	−0.2954 (2)	−0.04267 (18)	0.80687 (13)	0.0693 (8)	
C38	−0.2277 (2)	−0.09876 (17)	0.75726 (13)	0.0616 (8)	
C39	−0.2644 (2)	−0.1778 (2)	0.72150 (15)	0.0864 (10)	
C40	0.0380 (2)	0.05329 (15)	0.76010 (11)	0.0515 (7)	
C41	0.37427 (18)	0.04222 (13)	0.67934 (10)	0.0453 (6)	
C42	0.48957 (17)	0.10614 (13)	0.67331 (10)	0.0439 (6)	
C43	0.60845 (19)	0.09994 (14)	0.61589 (11)	0.0527 (7)	
C44	0.7161 (2)	0.15844 (16)	0.60846 (11)	0.0598 (7)	
C45	0.71458 (19)	0.22425 (14)	0.65804 (11)	0.0533 (7)	
C46	0.8259 (2)	0.28335 (17)	0.65383 (13)	0.0716 (8)	
C47	0.8210 (2)	0.34244 (17)	0.70435 (15)	0.0785 (10)	
C48	0.7061 (2)	0.34898 (17)	0.76139 (14)	0.0731 (9)	
C49	0.5965 (2)	0.29390 (15)	0.76774 (12)	0.0604 (7)	
C50	0.59774 (18)	0.22941 (13)	0.71683 (11)	0.0477 (6)	
C51	0.48799 (18)	0.16903 (13)	0.72286 (10)	0.0462 (6)	
H5B	1.19660	0.06780	0.51596	0.1222*	0.747 (3)
H5C	1.06783	0.06904	0.58243	0.1222*	0.747 (3)
H10	0.42122	0.39635	0.06919	0.0758*	
H12	0.44027	0.27554	−0.01927	0.0849*	
H13	0.53485	0.12481	−0.06021	0.0980*	
H14	0.68945	0.00942	−0.00428	0.0950*	
H15	0.75147	0.04645	0.09333	0.0764*	
H17	0.73341	0.16619	0.17963	0.0563*	
H6	0.84985	0.13503	0.37147	0.0649*	
H2A	0.97261	0.41395	0.37319	0.0759*	0.747 (3)
H2N	0.73033	0.18777	0.28734	0.0613*	
H3A	1.11989	0.36398	0.46372	0.0806*	0.747 (3)
H3O	0.50968	0.47994	0.19848	0.1381*	
H5A	1.18023	0.15569	0.56310	0.1222*	0.747 (3)
H2B	1.02185	0.06276	0.46423	0.1030*	0.253 (3)
H3B	1.12456	0.17477	0.52635	0.1030*	0.253 (3)
H5D	1.11452	0.46593	0.43825	0.1288*	0.253 (3)
H5E	1.08878	0.39962	0.52817	0.1288*	0.253 (3)
H5F	0.97134	0.45720	0.49329	0.1288*	0.253 (3)
H4N	0.80073	0.57443	0.20780	0.0618*	
H6O	0.94124	0.89522	0.03899	0.1186*	
H19	0.70270	0.41031	0.49193	0.0901*	
H20	0.60183	0.49106	0.60287	0.0945*	
H22A	0.52675	0.70863	0.60763	0.1597*	
H22B	0.47479	0.76850	0.53326	0.1597*	
H22C	0.61476	0.79722	0.53448	0.1597*	
H23	0.76231	0.50436	0.33077	0.0664*	
H27	0.99298	0.81971	−0.11979	0.0703*	
H29	0.98405	0.68880	−0.19749	0.0795*	
H30	0.92372	0.51929	−0.20043	0.0838*	
H31	0.82121	0.38089	−0.08550	0.0780*	
H32	0.77766	0.41586	0.03235	0.0658*	
H34	0.78453	0.54724	0.10822	0.0553*	
H6N	0.24982	0.12886	0.73705	0.0606*	
H9O	0.54982	0.00109	0.57882	0.1136*	
H36	−0.22506	0.07566	0.84606	0.0782*	
H37	−0.38499	−0.04805	0.83116	0.0831*	
H39A	−0.35821	−0.18535	0.73688	0.1295*	
H39B	−0.22443	−0.25144	0.73904	0.1295*	
H39C	−0.23452	−0.14793	0.66530	0.1295*	
H40	0.04078	0.11405	0.77906	0.0618*	
H44	0.79238	0.15481	0.56965	0.0717*	
H46	0.90363	0.28132	0.61559	0.0859*	
H47	0.89576	0.37939	0.70093	0.0940*	
H48	0.70407	0.39100	0.79526	0.0877*	
H49	0.51989	0.29873	0.80603	0.0724*	
H51	0.41136	0.17154	0.76172	0.0554*	

### Atomic displacement parameters (Å^2^)

**Table:**

	*U*^11^	*U*^22^	*U*^33^	*U*^12^	*U*^13^	*U*^23^
O1A	0.062 (2)	0.0574 (15)	0.0452 (13)	−0.0028 (14)	−0.0083 (13)	−0.0145 (12)
O2	0.0890 (10)	0.0427 (7)	0.0647 (9)	−0.0060 (6)	−0.0230 (8)	−0.0220 (6)
O3	0.1054 (13)	0.0801 (10)	0.1190 (14)	0.0425 (9)	−0.0531 (11)	−0.0613 (10)
N1	0.0606 (10)	0.0534 (9)	0.0515 (10)	−0.0088 (8)	−0.0150 (9)	−0.0219 (8)
N2	0.0667 (10)	0.0432 (8)	0.0508 (10)	−0.0109 (7)	−0.0196 (8)	−0.0180 (7)
C1A	0.0464 (18)	0.057 (2)	0.0424 (18)	0.0036 (18)	−0.0095 (14)	−0.0197 (19)
C2A	0.071 (3)	0.059 (2)	0.074 (3)	0.0093 (19)	−0.039 (3)	−0.024 (2)
C3A	0.073 (3)	0.066 (2)	0.076 (3)	0.004 (2)	−0.035 (3)	−0.0274 (19)
C4A	0.0472 (19)	0.075 (2)	0.047 (2)	0.0022 (16)	−0.0127 (18)	−0.0199 (18)
C5A	0.083 (2)	0.095 (2)	0.064 (2)	0.0103 (18)	−0.0319 (18)	−0.0133 (18)
C6	0.0601 (13)	0.0591 (11)	0.0469 (12)	−0.0063 (10)	−0.0103 (10)	−0.0230 (10)
C7	0.0553 (12)	0.0408 (9)	0.0439 (11)	−0.0110 (8)	−0.0042 (9)	−0.0136 (8)
C8	0.0510 (11)	0.0414 (9)	0.0422 (11)	−0.0067 (8)	−0.0070 (9)	−0.0132 (8)
C9	0.0569 (13)	0.0492 (10)	0.0654 (14)	0.0074 (9)	−0.0131 (11)	−0.0237 (10)
C10	0.0599 (13)	0.0643 (12)	0.0713 (15)	0.0098 (10)	−0.0285 (12)	−0.0224 (11)
C11	0.0554 (12)	0.0556 (10)	0.0511 (12)	−0.0067 (9)	−0.0145 (10)	−0.0143 (9)
C12	0.0808 (16)	0.0804 (14)	0.0614 (14)	−0.0020 (12)	−0.0337 (12)	−0.0224 (12)
C13	0.109 (2)	0.0920 (16)	0.0615 (15)	−0.0052 (15)	−0.0349 (15)	−0.0346 (13)
C14	0.1039 (19)	0.0793 (15)	0.0710 (16)	0.0092 (14)	−0.0291 (15)	−0.0432 (13)
C15	0.0791 (15)	0.0613 (11)	0.0597 (14)	0.0085 (10)	−0.0227 (12)	−0.0299 (10)
C16	0.0510 (12)	0.0468 (9)	0.0431 (11)	−0.0055 (8)	−0.0102 (9)	−0.0134 (8)
C17	0.0526 (12)	0.0421 (9)	0.0461 (11)	−0.0031 (8)	−0.0133 (9)	−0.0124 (8)
C1B	0.095 (5)	0.092 (4)	0.096 (5)	0.001 (3)	−0.040 (4)	−0.049 (4)
C2B	0.095 (5)	0.092 (4)	0.096 (5)	0.001 (3)	−0.040 (4)	−0.049 (4)
C3B	0.095 (5)	0.092 (4)	0.096 (5)	0.001 (3)	−0.040 (4)	−0.049 (4)
C4B	0.095 (5)	0.092 (4)	0.096 (5)	0.001 (3)	−0.040 (4)	−0.049 (4)
C5B	0.095 (5)	0.092 (4)	0.096 (5)	0.001 (3)	−0.040 (4)	−0.049 (4)
O1B	0.095 (5)	0.092 (4)	0.096 (5)	0.001 (3)	−0.040 (4)	−0.049 (4)
O4	0.0708 (9)	0.0619 (8)	0.0501 (9)	0.0040 (7)	−0.0119 (7)	−0.0201 (7)
O5	0.0981 (11)	0.0463 (7)	0.0701 (10)	−0.0098 (7)	−0.0129 (8)	−0.0283 (7)
O6	0.1093 (12)	0.0463 (7)	0.0755 (10)	−0.0209 (7)	−0.0129 (10)	−0.0145 (7)
N3	0.0586 (10)	0.0450 (8)	0.0495 (10)	0.0013 (7)	−0.0179 (8)	−0.0221 (8)
N4	0.0728 (11)	0.0406 (8)	0.0486 (10)	0.0017 (7)	−0.0177 (8)	−0.0232 (8)
C18	0.0678 (14)	0.0488 (10)	0.0531 (13)	−0.0021 (9)	−0.0245 (11)	−0.0140 (10)
C19	0.1016 (19)	0.0621 (13)	0.0624 (15)	−0.0051 (12)	−0.0329 (14)	−0.0090 (12)
C20	0.0849 (17)	0.0909 (17)	0.0506 (14)	−0.0115 (14)	−0.0202 (13)	−0.0028 (13)
C21	0.0616 (14)	0.0959 (17)	0.0458 (14)	−0.0006 (12)	−0.0091 (11)	−0.0224 (12)
C22	0.109 (2)	0.133 (2)	0.0724 (17)	0.0223 (18)	0.0000 (16)	−0.0515 (17)
C23	0.0738 (14)	0.0473 (10)	0.0560 (13)	0.0033 (9)	−0.0280 (11)	−0.0224 (10)
C24	0.0484 (11)	0.0426 (9)	0.0566 (12)	0.0022 (8)	−0.0160 (9)	−0.0214 (9)
C25	0.0449 (11)	0.0400 (9)	0.0487 (11)	0.0030 (8)	−0.0137 (9)	−0.0147 (8)
C26	0.0566 (12)	0.0407 (9)	0.0578 (13)	0.0002 (8)	−0.0142 (10)	−0.0120 (9)
C27	0.0638 (14)	0.0494 (10)	0.0489 (13)	−0.0021 (9)	−0.0083 (10)	−0.0018 (9)
C28	0.0512 (12)	0.0539 (10)	0.0460 (12)	0.0078 (9)	−0.0147 (10)	−0.0137 (9)
C29	0.0742 (15)	0.0729 (14)	0.0434 (13)	0.0049 (11)	−0.0108 (11)	−0.0123 (10)
C30	0.0820 (16)	0.0849 (15)	0.0491 (14)	0.0121 (13)	−0.0179 (12)	−0.0326 (12)
C31	0.0743 (15)	0.0703 (13)	0.0625 (15)	0.0044 (11)	−0.0229 (12)	−0.0341 (12)
C32	0.0632 (13)	0.0554 (10)	0.0490 (12)	−0.0049 (9)	−0.0131 (10)	−0.0204 (9)
C33	0.0430 (11)	0.0478 (9)	0.0423 (11)	0.0023 (8)	−0.0115 (9)	−0.0136 (9)
C34	0.0503 (11)	0.0440 (9)	0.0415 (11)	−0.0043 (8)	−0.0090 (9)	−0.0112 (8)
O7	0.0468 (8)	0.0645 (8)	0.0599 (9)	−0.0066 (6)	−0.0104 (7)	−0.0213 (7)
O8	0.0701 (9)	0.0503 (7)	0.0711 (9)	−0.0062 (6)	−0.0075 (7)	−0.0344 (7)
O9	0.0750 (10)	0.0908 (10)	0.0713 (10)	−0.0099 (8)	0.0016 (8)	−0.0534 (9)
N5	0.0502 (10)	0.0474 (8)	0.0564 (10)	−0.0027 (7)	−0.0155 (8)	−0.0196 (7)
N6	0.0520 (10)	0.0456 (8)	0.0614 (10)	−0.0039 (7)	−0.0140 (8)	−0.0262 (8)
C35	0.0531 (13)	0.0500 (10)	0.0484 (12)	0.0015 (9)	−0.0134 (10)	−0.0144 (9)
C36	0.0641 (15)	0.0645 (12)	0.0592 (14)	0.0064 (11)	−0.0083 (12)	−0.0172 (10)
C37	0.0468 (13)	0.0766 (14)	0.0680 (15)	−0.0013 (11)	−0.0059 (11)	−0.0076 (12)
C38	0.0462 (13)	0.0684 (12)	0.0628 (14)	−0.0086 (10)	−0.0141 (11)	−0.0078 (11)
C39	0.0676 (16)	0.0980 (17)	0.0987 (19)	−0.0230 (13)	−0.0252 (14)	−0.0277 (15)
C40	0.0569 (13)	0.0490 (10)	0.0517 (12)	0.0027 (9)	−0.0185 (10)	−0.0169 (9)
C41	0.0540 (12)	0.0379 (8)	0.0441 (11)	0.0003 (8)	−0.0119 (9)	−0.0137 (8)
C42	0.0512 (12)	0.0373 (8)	0.0436 (11)	0.0018 (8)	−0.0119 (9)	−0.0139 (8)
C43	0.0604 (13)	0.0510 (10)	0.0471 (12)	−0.0013 (9)	−0.0068 (10)	−0.0215 (9)
C44	0.0560 (13)	0.0648 (12)	0.0518 (13)	−0.0074 (10)	0.0017 (10)	−0.0204 (10)
C45	0.0546 (13)	0.0463 (10)	0.0537 (12)	−0.0045 (9)	−0.0103 (10)	−0.0103 (9)
C46	0.0600 (14)	0.0670 (13)	0.0816 (16)	−0.0117 (11)	−0.0075 (12)	−0.0207 (12)
C47	0.0704 (17)	0.0668 (13)	0.109 (2)	−0.0104 (11)	−0.0305 (15)	−0.0327 (14)
C48	0.0719 (16)	0.0707 (13)	0.0986 (19)	0.0059 (12)	−0.0352 (15)	−0.0466 (13)
C49	0.0590 (13)	0.0609 (11)	0.0733 (14)	0.0073 (10)	−0.0225 (11)	−0.0347 (11)
C50	0.0530 (12)	0.0401 (9)	0.0531 (12)	0.0035 (8)	−0.0186 (10)	−0.0156 (8)
C51	0.0471 (11)	0.0446 (9)	0.0474 (11)	0.0065 (8)	−0.0113 (9)	−0.0179 (8)

### Geometric parameters (Å, °)

**Table:**

O1A—C1A	1.363 (7)	C12—H12	0.9300
O1A—C4A	1.364 (9)	C13—H13	0.9300
O1B—C1B	1.38 (3)	C14—H14	0.9300
O1B—C4B	1.37 (3)	C15—H15	0.9300
O2—C7	1.243 (2)	C17—H17	0.9300
O3—C9	1.354 (3)	C18—C19	1.347 (3)
O3—H3O	0.8200	C18—C23	1.425 (3)
O4—C21	1.372 (3)	C19—C20	1.402 (3)
O4—C18	1.375 (2)	C20—C21	1.332 (4)
O5—C24	1.235 (2)	C21—C22	1.479 (4)
O6—C26	1.353 (2)	C24—C25	1.482 (3)
O6—H6O	0.8200	C25—C26	1.428 (3)
O7—C35	1.369 (2)	C25—C34	1.380 (3)
O7—C38	1.378 (3)	C26—C27	1.365 (3)
O8—C41	1.242 (2)	C27—C28	1.406 (3)
O9—C43	1.363 (3)	C28—C29	1.420 (3)
O9—H9O	0.8200	C28—C33	1.414 (3)
N1—N2	1.373 (2)	C29—C30	1.350 (3)
N1—C6	1.273 (3)	C30—C31	1.400 (3)
N2—C7	1.338 (2)	C31—C32	1.358 (3)
N2—H2N	0.8600	C32—C33	1.410 (3)
N3—C23	1.281 (3)	C33—C34	1.406 (3)
N3—N4	1.388 (2)	C19—H19	0.9300
N4—C24	1.343 (2)	C20—H20	0.9300
N4—H4N	0.8600	C22—H22C	0.9600
N5—C40	1.278 (3)	C22—H22B	0.9600
N5—N6	1.387 (2)	C22—H22A	0.9600
N6—C41	1.337 (3)	C23—H23	0.9300
N6—H6N	0.8600	C27—H27	0.9300
C1A—C6	1.442 (6)	C29—H29	0.9300
C1A—C2A	1.323 (7)	C30—H30	0.9300
C1B—C2B	1.32 (4)	C31—H31	0.9300
C1B—C6	1.42 (3)	C32—H32	0.9300
C2A—C3A	1.405 (8)	C34—H34	0.9300
C2B—C3B	1.44 (4)	C35—C40	1.423 (3)
C3A—C4A	1.340 (9)	C35—C36	1.346 (3)
C3B—C4B	1.32 (4)	C36—C37	1.411 (3)
C4A—C5A	1.461 (8)	C37—C38	1.336 (3)
C4B—C5B	1.45 (2)	C38—C39	1.476 (3)
C7—C8	1.474 (3)	C41—C42	1.479 (3)
C8—C9	1.425 (3)	C42—C43	1.420 (3)
C8—C17	1.382 (2)	C42—C51	1.377 (2)
C9—C10	1.365 (3)	C43—C44	1.362 (3)
C10—C11	1.398 (3)	C44—C45	1.406 (3)
C11—C16	1.418 (3)	C45—C50	1.417 (3)
C11—C12	1.417 (3)	C45—C46	1.419 (3)
C12—C13	1.351 (4)	C46—C47	1.349 (3)
C13—C14	1.391 (4)	C47—C48	1.389 (3)
C14—C15	1.355 (3)	C48—C49	1.362 (3)
C15—C16	1.412 (3)	C49—C50	1.415 (3)
C16—C17	1.401 (3)	C50—C51	1.402 (3)
C2A—H2A	0.9300	C36—H36	0.9300
C2B—H2B	0.9300	C37—H37	0.9300
C3A—H3A	0.9300	C39—H39A	0.9600
C3B—H3B	0.9300	C39—H39B	0.9600
C5A—H5B	0.9600	C39—H39C	0.9600
C5A—H5A	0.9600	C40—H40	0.9300
C5A—H5C	0.9600	C44—H44	0.9300
C5B—H5D	0.9600	C46—H46	0.9300
C5B—H5E	0.9600	C47—H47	0.9300
C5B—H5F	0.9600	C48—H48	0.9300
C6—H6	0.9300	C49—H49	0.9300
C10—H10	0.9300	C51—H51	0.9300
			
C1A—O1A—C4A	107.6 (5)	N3—C23—C18	124.41 (18)
C1B—O1B—C4B	109.4 (19)	O5—C24—C25	121.60 (17)
C9—O3—H3O	109.00	O5—C24—N4	120.42 (17)
C18—O4—C21	106.72 (16)	N4—C24—C25	117.98 (16)
C26—O6—H6O	109.00	C24—C25—C26	119.01 (16)
C35—O7—C38	106.53 (15)	C26—C25—C34	117.86 (16)
C43—O9—H9O	109.00	C24—C25—C34	123.13 (16)
N2—N1—C6	114.76 (16)	C25—C26—C27	119.91 (17)
N1—N2—C7	118.84 (15)	O6—C26—C27	117.64 (18)
C7—N2—H2N	121.00	O6—C26—C25	122.46 (17)
N1—N2—H2N	121.00	C26—C27—C28	122.19 (18)
N4—N3—C23	113.59 (15)	C29—C28—C33	117.99 (18)
N3—N4—C24	119.83 (15)	C27—C28—C29	123.13 (18)
C24—N4—H4N	120.00	C27—C28—C33	118.88 (17)
N3—N4—H4N	120.00	C28—C29—C30	120.89 (19)
N6—N5—C40	114.51 (15)	C29—C30—C31	121.2 (2)
N5—N6—C41	119.07 (14)	C30—C31—C32	119.6 (2)
N5—N6—H6N	120.00	C31—C32—C33	121.08 (18)
C41—N6—H6N	120.00	C28—C33—C32	119.25 (16)
C2A—C1A—C6	135.8 (5)	C28—C33—C34	118.02 (17)
O1A—C1A—C6	114.8 (4)	C32—C33—C34	122.73 (16)
O1A—C1A—C2A	109.3 (5)	C25—C34—C33	123.12 (16)
C2B—C1B—C6	138 (2)	C18—C19—H19	126.00
O1B—C1B—C6	114 (2)	C20—C19—H19	126.00
O1B—C1B—C2B	108 (2)	C19—C20—H20	126.00
C1A—C2A—C3A	107.2 (5)	C21—C20—H20	126.00
C1B—C2B—C3B	107 (2)	H22A—C22—H22C	109.00
C2A—C3A—C4A	107.5 (5)	C21—C22—H22A	110.00
C2B—C3B—C4B	108 (3)	C21—C22—H22B	109.00
C3A—C4A—C5A	135.6 (7)	H22A—C22—H22B	109.00
O1A—C4A—C3A	108.3 (6)	H22B—C22—H22C	109.00
O1A—C4A—C5A	116.0 (6)	C21—C22—H22C	110.00
C3B—C4B—C5B	136 (2)	N3—C23—H23	118.00
O1B—C4B—C5B	116.6 (18)	C18—C23—H23	118.00
O1B—C4B—C3B	107 (2)	C26—C27—H27	119.00
N1—C6—C1B	130.3 (9)	C28—C27—H27	119.00
N1—C6—C1A	116.7 (2)	C28—C29—H29	120.00
N2—C7—C8	117.68 (16)	C30—C29—H29	120.00
O2—C7—N2	121.55 (17)	C29—C30—H30	119.00
O2—C7—C8	120.73 (17)	C31—C30—H30	119.00
C9—C8—C17	118.17 (16)	C32—C31—H31	120.00
C7—C8—C9	119.94 (16)	C30—C31—H31	120.00
C7—C8—C17	121.53 (17)	C33—C32—H32	119.00
O3—C9—C10	118.59 (19)	C31—C32—H32	119.00
O3—C9—C8	120.92 (18)	C25—C34—H34	118.00
C8—C9—C10	120.49 (18)	C33—C34—H34	118.00
C9—C10—C11	121.44 (19)	O7—C35—C40	120.29 (17)
C12—C11—C16	117.87 (18)	C36—C35—C40	130.21 (18)
C10—C11—C12	123.03 (19)	O7—C35—C36	109.49 (18)
C10—C11—C16	119.08 (18)	C35—C36—C37	107.10 (19)
C11—C12—C13	120.6 (2)	C36—C37—C38	107.2 (2)
C12—C13—C14	121.5 (2)	O7—C38—C37	109.63 (19)
C13—C14—C15	120.1 (2)	O7—C38—C39	116.42 (18)
C14—C15—C16	120.5 (2)	C37—C38—C39	133.9 (2)
C11—C16—C17	118.68 (16)	N5—C40—C35	122.94 (18)
C11—C16—C15	119.50 (17)	N6—C41—C42	117.24 (15)
C15—C16—C17	121.81 (18)	O8—C41—N6	121.25 (18)
C8—C17—C16	122.13 (17)	O8—C41—C42	121.51 (17)
C3A—C2A—H2A	126.00	C41—C42—C43	119.03 (15)
C1A—C2A—H2A	126.00	C41—C42—C51	122.69 (16)
C3B—C2B—H2B	127.00	C43—C42—C51	118.25 (17)
C1B—C2B—H2B	126.00	C42—C43—C44	120.47 (17)
C2A—C3A—H3A	126.00	O9—C43—C42	121.35 (18)
C4A—C3A—H3A	126.00	O9—C43—C44	118.18 (18)
C2B—C3B—H3B	126.00	C43—C44—C45	121.56 (19)
C4B—C3B—H3B	126.00	C44—C45—C46	123.23 (19)
C4A—C5A—H5C	109.00	C44—C45—C50	118.78 (18)
H5B—C5A—H5C	110.00	C46—C45—C50	117.97 (17)
H5A—C5A—H5C	109.00	C45—C46—C47	121.1 (2)
C4A—C5A—H5A	109.00	C46—C47—C48	121.1 (2)
H5A—C5A—H5B	109.00	C47—C48—C49	120.0 (2)
C4A—C5A—H5B	109.00	C48—C49—C50	120.8 (2)
C4B—C5B—H5E	110.00	C45—C50—C49	118.96 (18)
H5D—C5B—H5E	110.00	C45—C50—C51	118.52 (16)
C4B—C5B—H5D	110.00	C49—C50—C51	122.52 (18)
H5E—C5B—H5F	109.00	C42—C51—C50	122.39 (17)
C4B—C5B—H5F	109.00	C35—C36—H36	126.00
H5D—C5B—H5F	109.00	C37—C36—H36	126.00
C1A—C6—H6	122.00	C36—C37—H37	126.00
N1—C6—H6	122.00	C38—C37—H37	126.00
C1B—C6—H6	108.00	C38—C39—H39A	109.00
C11—C10—H10	119.00	C38—C39—H39B	109.00
C9—C10—H10	119.00	C38—C39—H39C	109.00
C13—C12—H12	120.00	H39A—C39—H39B	109.00
C11—C12—H12	120.00	H39A—C39—H39C	109.00
C14—C13—H13	119.00	H39B—C39—H39C	110.00
C12—C13—H13	119.00	N5—C40—H40	119.00
C15—C14—H14	120.00	C35—C40—H40	119.00
C13—C14—H14	120.00	C43—C44—H44	119.00
C14—C15—H15	120.00	C45—C44—H44	119.00
C16—C15—H15	120.00	C45—C46—H46	119.00
C8—C17—H17	119.00	C47—C46—H46	119.00
C16—C17—H17	119.00	C46—C47—H47	119.00
O4—C18—C23	120.66 (17)	C48—C47—H47	119.00
C19—C18—C23	130.56 (19)	C47—C48—H48	120.00
O4—C18—C19	108.63 (18)	C49—C48—H48	120.00
C18—C19—C20	107.7 (2)	C48—C49—H49	120.00
C19—C20—C21	107.2 (2)	C50—C49—H49	120.00
O4—C21—C22	116.5 (2)	C42—C51—H51	119.00
O4—C21—C20	109.8 (2)	C50—C51—H51	119.00
C20—C21—C22	133.7 (2)		
			
C4A—O1A—C1A—C2A	0.9 (7)	C19—C20—C21—O4	−0.9 (3)
C4A—O1A—C1A—C6	177.9 (5)	C19—C20—C21—C22	177.8 (3)
C1A—O1A—C4A—C3A	−0.3 (7)	N4—C24—C25—C34	13.2 (3)
C1A—O1A—C4A—C5A	176.8 (5)	O5—C24—C25—C26	13.1 (3)
C21—O4—C18—C19	−0.5 (2)	O5—C24—C25—C34	−167.32 (19)
C21—O4—C18—C23	175.60 (19)	N4—C24—C25—C26	−166.32 (17)
C18—O4—C21—C20	0.9 (2)	C24—C25—C26—C27	178.46 (18)
C18—O4—C21—C22	−178.1 (2)	C34—C25—C26—O6	179.23 (18)
C35—O7—C38—C37	−0.9 (2)	C34—C25—C26—C27	−1.1 (3)
C35—O7—C38—C39	177.11 (18)	C24—C25—C34—C33	−179.04 (18)
C38—O7—C35—C36	0.2 (2)	C26—C25—C34—C33	0.5 (3)
C38—O7—C35—C40	179.94 (18)	C24—C25—C26—O6	−1.2 (3)
N2—N1—C6—C1A	174.2 (3)	O6—C26—C27—C28	−179.11 (19)
C6—N1—N2—C7	−179.29 (17)	C25—C26—C27—C28	1.2 (3)
N1—N2—C7—C8	167.77 (15)	C26—C27—C28—C29	179.2 (2)
N1—N2—C7—O2	−9.9 (3)	C26—C27—C28—C33	−0.7 (3)
N4—N3—C23—C18	−174.98 (19)	C27—C28—C29—C30	−179.7 (2)
C23—N3—N4—C24	−175.09 (18)	C33—C28—C29—C30	0.2 (3)
N3—N4—C24—O5	5.5 (3)	C27—C28—C33—C32	−179.56 (19)
N3—N4—C24—C25	−175.01 (16)	C27—C28—C33—C34	0.0 (3)
C40—N5—N6—C41	−177.91 (17)	C29—C28—C33—C32	0.6 (3)
N6—N5—C40—C35	178.16 (17)	C29—C28—C33—C34	−179.85 (18)
N5—N6—C41—O8	0.9 (3)	C28—C29—C30—C31	−0.7 (3)
N5—N6—C41—C42	−179.52 (15)	C29—C30—C31—C32	0.4 (3)
O1A—C1A—C2A—C3A	−1.1 (6)	C30—C31—C32—C33	0.4 (3)
C6—C1A—C2A—C3A	−177.2 (6)	C31—C32—C33—C28	−0.8 (3)
C2A—C1A—C6—N1	−9.9 (8)	C31—C32—C33—C34	179.6 (2)
O1A—C1A—C6—N1	174.2 (4)	C28—C33—C34—C25	0.0 (3)
C1A—C2A—C3A—C4A	0.9 (7)	C32—C33—C34—C25	179.59 (19)
C2A—C3A—C4A—C5A	−176.6 (7)	O7—C35—C36—C37	0.5 (2)
C2A—C3A—C4A—O1A	−0.3 (8)	C40—C35—C36—C37	−179.2 (2)
O2—C7—C8—C9	−17.6 (3)	O7—C35—C40—N5	−9.8 (3)
N2—C7—C8—C17	−22.3 (3)	C36—C35—C40—N5	170.0 (2)
N2—C7—C8—C9	164.73 (17)	C35—C36—C37—C38	−1.0 (3)
O2—C7—C8—C17	155.33 (18)	C36—C37—C38—O7	1.2 (2)
C7—C8—C9—O3	−5.2 (3)	C36—C37—C38—C39	−176.3 (2)
C7—C8—C9—C10	174.57 (18)	O8—C41—C42—C43	17.5 (3)
C7—C8—C17—C16	−173.73 (17)	O8—C41—C42—C51	−160.42 (17)
C17—C8—C9—O3	−178.42 (18)	N6—C41—C42—C43	−162.12 (16)
C17—C8—C9—C10	1.4 (3)	N6—C41—C42—C51	20.0 (3)
C9—C8—C17—C16	−0.7 (3)	C41—C42—C43—O9	−0.2 (3)
C8—C9—C10—C11	−1.4 (3)	C41—C42—C43—C44	179.53 (17)
O3—C9—C10—C11	178.38 (19)	C51—C42—C43—O9	177.80 (17)
C9—C10—C11—C12	−177.7 (2)	C51—C42—C43—C44	−2.5 (3)
C9—C10—C11—C16	0.7 (3)	C41—C42—C51—C50	179.96 (17)
C12—C11—C16—C17	178.51 (18)	C43—C42—C51—C50	2.0 (3)
C10—C11—C16—C15	−178.63 (19)	O9—C43—C44—C45	−178.71 (18)
C10—C11—C16—C17	0.0 (3)	C42—C43—C44—C45	1.5 (3)
C12—C11—C16—C15	−0.2 (3)	C43—C44—C45—C46	177.9 (2)
C16—C11—C12—C13	0.1 (3)	C43—C44—C45—C50	−0.1 (3)
C10—C11—C12—C13	178.5 (2)	C44—C45—C46—C47	−177.8 (2)
C11—C12—C13—C14	0.1 (4)	C50—C45—C46—C47	0.2 (3)
C12—C13—C14—C15	−0.3 (4)	C44—C45—C50—C49	178.90 (18)
C13—C14—C15—C16	0.2 (4)	C44—C45—C50—C51	−0.3 (3)
C14—C15—C16—C17	−178.6 (2)	C46—C45—C50—C49	0.8 (3)
C14—C15—C16—C11	0.0 (3)	C46—C45—C50—C51	−178.44 (18)
C11—C16—C17—C8	0.0 (3)	C45—C46—C47—C48	−1.1 (4)
C15—C16—C17—C8	178.60 (18)	C46—C47—C48—C49	0.9 (4)
C23—C18—C19—C20	−175.6 (2)	C47—C48—C49—C50	0.1 (3)
O4—C18—C23—N3	9.4 (3)	C48—C49—C50—C45	−1.0 (3)
O4—C18—C19—C20	−0.1 (2)	C48—C49—C50—C51	178.27 (19)
C19—C18—C23—N3	−175.5 (2)	C45—C50—C51—C42	−0.7 (3)
C18—C19—C20—C21	0.6 (3)	C49—C50—C51—C42	−179.87 (18)

### Hydrogen-bond geometry (Å, °)

**Table:**

*D*—H···*A*	*D*—H	H···*A*	*D*···*A*	*D*—H···*A*
N2—H2N···O8^i^	0.86	2.15	2.869 (2)	142
N4—H4N···O2	0.86	2.20	2.896 (2)	137
N6—H6N···O5^ii^	0.86	2.36	3.074 (2)	140
N6—H6N···N3^ii^	0.86	2.44	3.212 (2)	149
O3—H3O···O2	0.82	1.89	2.612 (3)	145
O6—H6O···O5	0.82	1.89	2.603 (2)	145
O9—H9O···O8	0.82	1.86	2.588 (2)	147
C34—H34···O2	0.93	2.48	3.398 (3)	167
C40—H40···O5^ii^	0.93	2.42	3.185 (3)	140
C44—H44···O1A	0.93	2.59	3.514 (5)	176
C48—H48···O3^ii^	0.93	2.51	3.149 (3)	126

Symmetry codes: (i) −*x*+1, −*y*, −*z*+1; (ii) −*x*+1, −*y*+1, −*z*+1.
